# Identification and Documentation of Auricle Defects using Three-dimensional Optical Measurements

**DOI:** 10.1038/s41598-018-21289-x

**Published:** 2018-02-12

**Authors:** Guomin Zhan, Liya Han, Zhongwei Li, Zilong Liu, Jiaqi Fu, Kai Zhong

**Affiliations:** 1 State Key Laboratory of Material Processing and Die & Mould Technology, Huazhong University of Science and Technology, Wuhan, 430074 China; 2 Department of Forensic Medicine, Tongji Medical College of Huazhong University of Science and Technology, Wuhan, 430074 China

## Abstract

Auricle defects are important and common occurrences in forensic medicine. The accurate measurement and assessment of auricle defects is key to identifying and evaluating injury, and the currently available methods are known to be labor intensive and inaccurate. In this paper, we introduce an identification and documentation of auricle defects solution, which consists of an optical three-dimensional (3D) method and an effective algorithm to calculate the maximum projection area and identify auricle defects. In this study, three separate examiners measured 40 auricles of 20 adults using 3D optical measurement and two other commonly used methods to investigate the validity and representative reliability of 3D optical measurement for auricle defect identification. Based on the statistical analysis, the 3D measurement method is valid and showed a better reliability than the reference methods. We also present a representative auricle defect identification case using the proposed 3D optical measurement method. The study concludes that the optical 3D measurement method is a reliable and effective tool for auricle defect identification.

## Introduction

Auricle defects caused by trauma, burns, freezing, piercing, or infection are important and common cases in clinical forensic medicine practice. The World Health Organization and the laws of many countries use defect percentiles as the standard for functioning and disability classification^1^. In order to identify the auricle defects, the literature often use the maximum projection ratio of the defective to heathy auricle areas to estimate the percentage of defects^2^.

To get the maximum projection ratio of the defective to heathy auricle areas, several feasible techniques have been proposed recently, such as Radiological Body Volume Documentation (Computed Tomography and Magnetic Resonance Imaging)^2–4^, and the two other planimetry methods (tracing planimetry and digital camera photogrammetry planimetry)^5–10^. Some papers reported the auricle 3d reconstruction by CT^2–4^, but due to a limited radiation exposure of living persons, scans in the clinical environment are normally not of such a high slice resolution^11^ (Typical value: CT 1.25 mm; MRI 4 mm). Because of the irregular shape of auricle, the low resolution would cause the results in a low accuracy. In the meanwhile, radiological imaging technologies are usually expensive, poor portability and exist radioactive. The planimetry methods are commonly used in forensic practices. Tracing planimetry consists of manually tracing the auricle on transparent grid paper^5,6^ and summing over all the traced grids. The second method is digital camera photogrammetry planimetry, in which an auricle is manually photographed and transferred into image-processing software to determine the defect profile and calculate the areas based on standard fixed area grid paper for scale^7–10^. Tracing the auricle onto a transparent sheet may be subject to difficulty in fixating the sheet and determining the irregular boundaries of the auricle through the transparent sheet. Furthermore, digital camera photogrammetry is not accurate enough for auricle defect measurement, because the viewing angle of the image taken by each rater is not exactly the same and lens nonlinear distortion also causes errors^12^. While the two reference methods have obvious shortcomings as mentioned above, they are still widely used because of their high practicality in forensic investigations.

In this paper, we propose 3D optical measurement method to solve these problems, this method acquire 3D information from a set of image pairs, which contain the illumination of phase shifting pattern modulated by measured object. Optical measurement method has a high measure accuracy of 0.02 mm and a high spatial resolution of 0.1 mm in all directions. This method is non-contact, non-radiative, low-cost and friendly to the patients. According to these aspects, we think the 3D optical method have the potential to identify the auricle defects.

As optical 3D measurement methods have developed gradually, they have increasingly been applied in forensic medicine^13^, especially for bite mark analysis^14,15^ and traffic accident analysis^16^. The article^11,16^ have reported the combination application of optical body surface and radiological CT/MRI internal body scanning. These applications also show the great potential and advantages of optical 3D measurement methods in forensic medicine. To the best of our knowledge, existing optical 3D measurement technologies have never been directly applied to auricle defect measurement because: (1) In practical forensic medicine, patients cannot keep absolutely still during the measurement. Hence, we need to implement a high-speed and highly accurate optical 3D measurement method for auricle defects. (2) After the 3D measurement data have been acquired, it is hard to determine the projection plane that obtains the maximum projection area. Manual operations are not accurate enough and are time consuming. Hence, we need an automatic and accurate algorithm to determine the maximum projection area.

In the present study, we apply multi-view phase shifting principle to 3D surface scanning, making the measurement complete in 0.1 s. A reliable and efficient algorithm is proposed to calculate the maximum projection areas of point clouds (massive 3D coordinates of surface points). The auricle surface areas can be calculated as a reference in the process. We measured 40 auricles of 20 adults using 3D optical measurement and the other two previously mentioned planimetry methods, and all measurements were repeated after several days by three additional examiners to investigate the validity and representative reliability of 3D optical measurement for auricle defect identification. We also present a representative auricle defect identification case using the proposed 3D optical measurement method.

## Materials and Methods

For the study, the normal auricles of 20 adults were measured to determine the auricle maximum projection areas using the three methods by three examiners at different times. The subjects were all Chinese, and consisted of nine female and 11 male individuals. The average age of the female subjects was 26.1 years (SD = 1.6), and that of the male subjects was 25.9 years (SD = 1.7). The auricle of each subject was digitized by the optical 3D measurement method to obtain point cloud data. We then reconstructed the surface (via triangulation) in Geomagic Studio (www.geomagic.com), and calculated the surface area. We also found the maximum projection area using an algorithm from the Point Cloud Library (PCL) (www.pointclouds.org, PCL 1.7). The other reference methods are detailed in the following sections.

The study protocol was approved by the Ethics Committee of Tongji Medical College, Huazhong University of Science and Technology (IORG No: IORG0003571). All experiments were performed in accordance with ICH-GCP, GCP in China and the Declaration of Helsinki. Informed consent was obtained from the participants after they had been given an explanation of the study. The manuscript reporting a participant’s details state that consent to publication was obtained from the patient.

### 3D surface scanning

**Figure 1:**
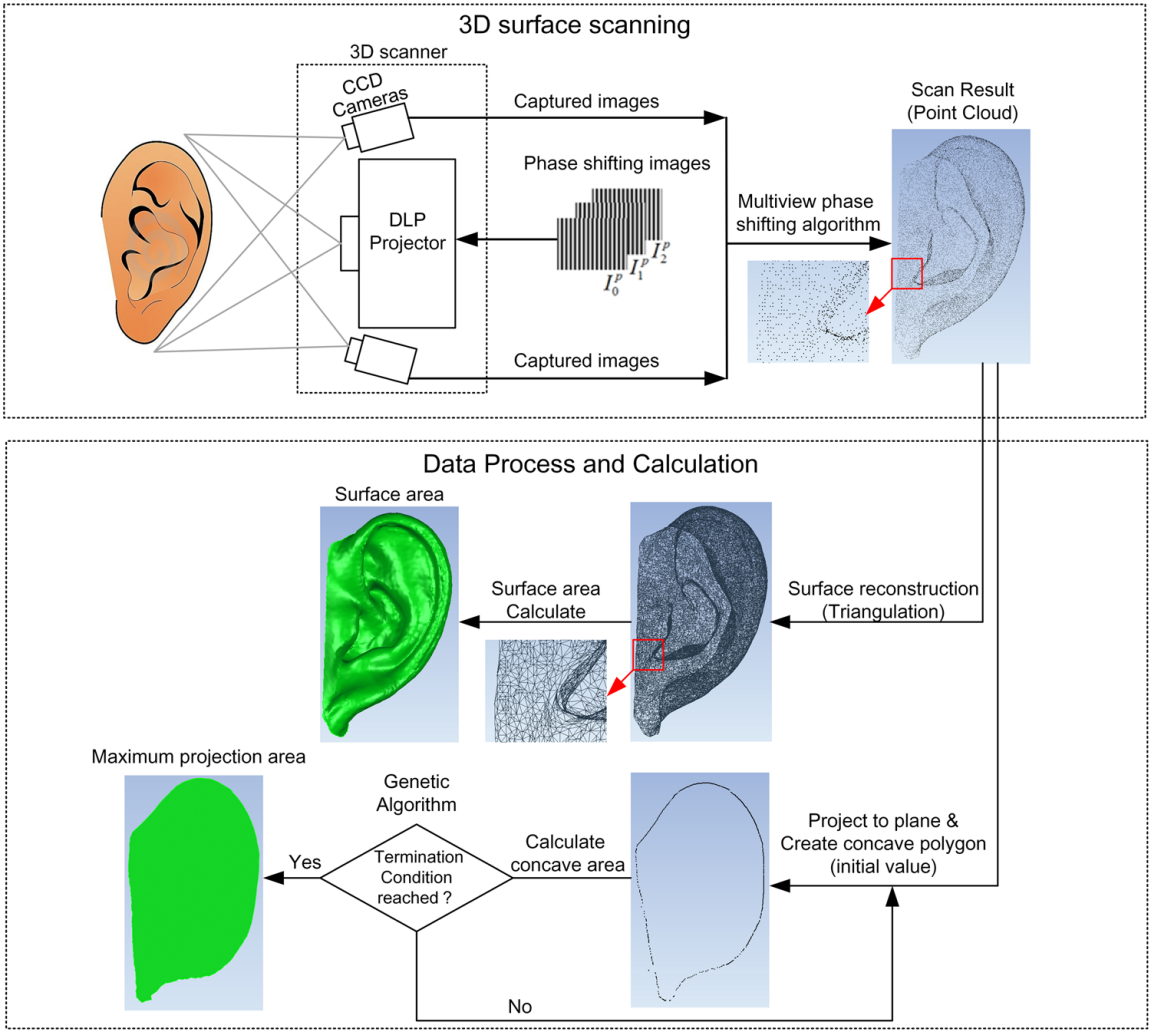
3D optical measurement method.

The 3D measurement of the auricle was performed using our structured light system, a complete description and validation of which was presented in^18^. Figure 1 shows the general framework of our 3D optical measurement system. Briefly, phase shifting images (${I}_{0}^{p},{I}_{1}^{p},{I}_{2}^{p}$) are projected sequentially at 30 frames/s from a DLP projector (DLP LightCrafter 4500, TI, Dallas, Texas, USA) onto an object to produce sinusoidal fringe patterns, which are recorded by two CCD cameras (acA1300-30gm; Basler, Ahrensburg, Germany) mounted on both sides of the projector.

Common 3D surface scanners are usually used for examination of industrial products, it takes several seconds for a single measurement. It works well with static objects but faces difficulties when applied in measurement of human body part. The slight movements of body would bring much noise in measurement results. The measurement speed need to be improved to satisfy the requirements of auricle defects measurement. In the current study, the multi-view phase shifting 3D measurement principle we proposed in^18^ was applied. This principle reduces the number of projected patterns to three. Meanwhile, to improve the transmission speed of the camera data, we divide the interframe time into two equal periods. In each period, only one camera occupies the bandwidth of the cable, which makes the transmission more reliable and reaches a higher frame rate. In this condition, a single measurement was completed within 0.1 s. Each measurement can calculate the high-precision 3D coordinates of up to a million surface points, the calculation time is about 1.5 s at the computer platform (Intel Core i7-4770K CPU, and 8 GB DDR3 1600 SDRAM, Opencv 2.4.2). The system has been calibrated by the method of Zhang^12^, the precision of this system is 0.02 mm, which was evaluated according to the Optical 3D Measuring Systems Standard (VDI/VDE 2634,^19^).

**Figure 2:**
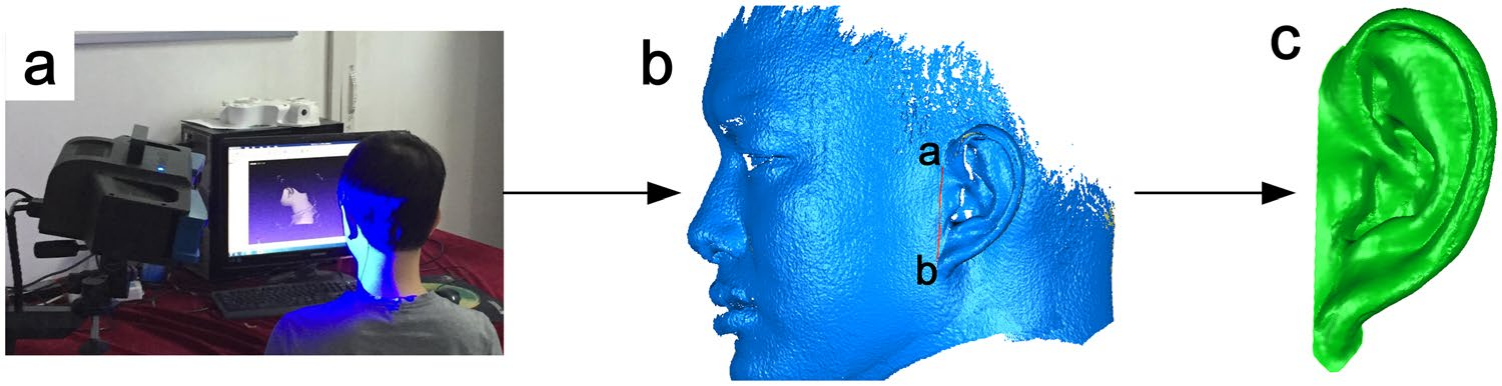
The work flow of 3D surface scanning. (**a**) Measurement scene. (**b**) Registration result and segment edge. (**c**) 3D surface scanning result.

As seen in Fig. 2a, the subject sits in front of the scanner at a distance of about 0.5 m, keeping the body still during the single scanning process. To scan the auricle in this study, the system scan range was adjusted to 200 × 160 mm2 with a resulting point spacing of 0.15 mm.

The auricle of the subject is measured from different directions to obtain the complete 3D auricle data. The point clouds from different directions are registered together based on the feature points of the auricle. The registration result is shown in Fig. 2b (Fig. S1).

Our application uses a semi-automatic process for auricle edge identification. We defined a line Fig. 2b from point a (the crus of the helix) to point b (the bottom of the auricle lobule) as the boundary between the auricle and face. Finally, we obtain the entire auricle point cloud Fig. 2c to use for the following processing and calculations.

### Data process and calculation

#### Surface reconstruction and surface area calculation

After obtaining the point cloud of the entire auricle, we reconstruct the surface (via triangulation) in Geomagic Studio using greedy projection^20^, which creates polygon meshes. For the occlusion caused by the complex structure of the auricle, some areas are invisible for the scanning system (shown as blank spots in Fig. 2(b)). And we noticed that these blind spots are distributed within the central area of the auricle, which has little influence on the maximum projected area calculation processes. Hence, we used the hole filling method^21^ to make the surface a closed region. The surface area is the sum of all the triangular areas, and is also calculated in Geomagic Studio.

#### Maximum projection area solution

**Figure 3:**
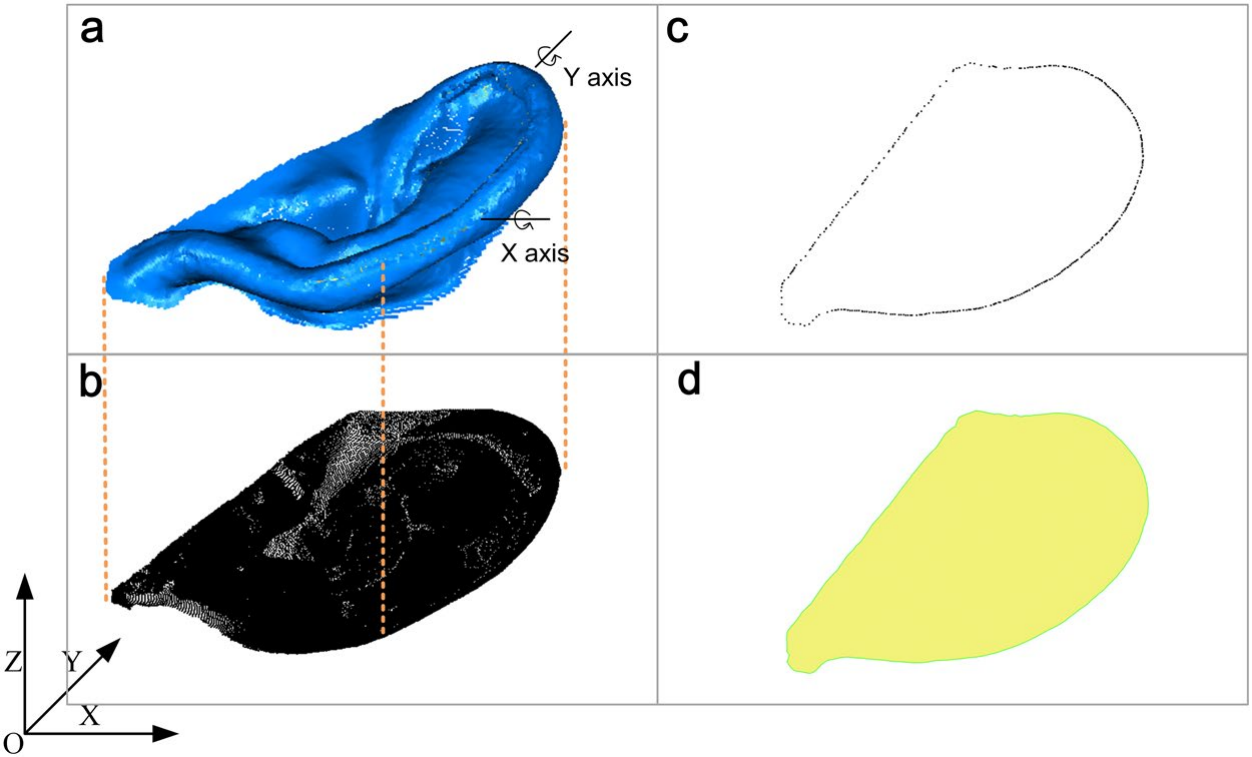
Projection area solution based on point cloud. (**a**) The point cloud of auricle. (**b**) The point cloud project to XOY plane. (**c**) The outer boundary points of projected points. (**d**) Concave polygon created by boundary points.

The maximum projection area problem is not defined in the area processes for industrial purpose, so we developed a maximum projection area solution. With this solution, the point cloud of the auricle can also be used to calculate the maximum projection area. As Fig. 3 shows, if the point cloud and its position are fixed, we can calculate the projection area using the following steps: (1) Project the point cloud onto the XOY plane (Fig. 3a,b). (2) Create a concave hull representation of the projected inliers using the PCL (Fig. 3c). (3) Calculate the concave polygon area (Fig. 3d). We can calculate the concave polygon area according to the following equation (1)^22^.

1 $${S}_{p}=\sum _{n=1}^{N}({p}_{i.x}{p}_{(i+1).y}-{p}_{i.y}{p}_{(i+1).x})+({p}_{N.x}{p}_{1.y}-{p}_{N.y}{p}_{1.x})$$

where *N* is the number of outer boundary points, *p*_*i,x*_ *p*_*i,y*_ are the coordinates of the *i* th point, and *S*_*p*_ is the area of the concave polygon.

To determine the maximum projection area, the variables are X-rotation (*φ*, pitch angle) and Y-rotation (*θ*, yaw angle). Optimization of both variables and maximization of the projection area can be efficiently expressed in the standard mathematical format as follows:

Find: *φ*, *θ*

Maximize: *S*_*p*_ = (*φ*,*θ*)

Within variable ranges: $-\frac{\pi }{2} < \phi \le \frac{\pi }{2},-\frac{\pi }{2} < \theta \le \frac{\pi }{2}$

**Figure 4:**
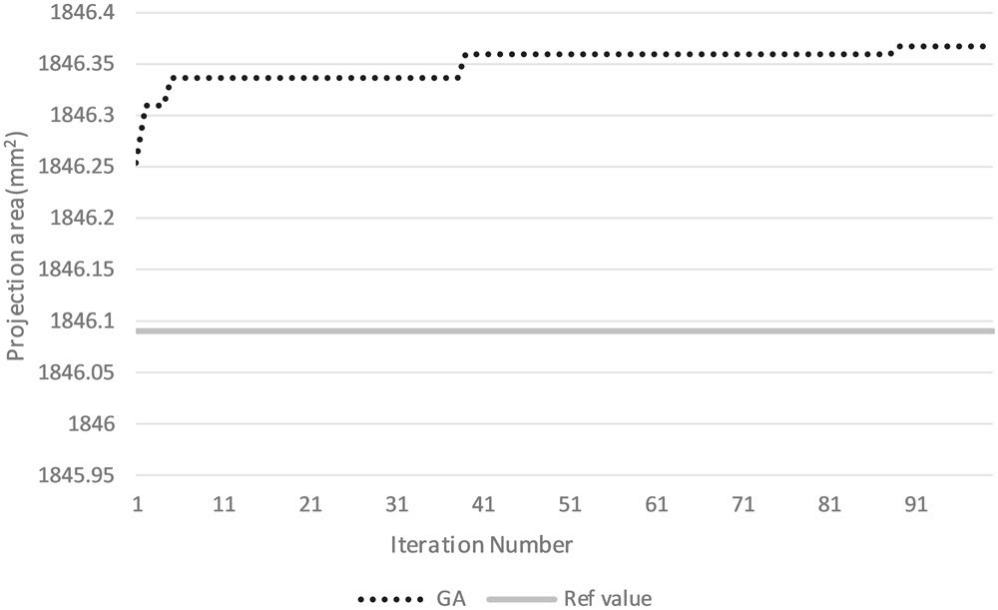
Maximization history with iterations for projection area.

To solve the maximization problem in equation (1), an effective genetic algorithm (GA) was developed and used in this study^23^. The critical parameters of a GA are the size of the population, mutation rate, and the number of iterations (i.e., generations). In this study, we employed a population size of 50, crossover rate of 0.8, mutation rate of 0.05, bit number for each variable of 16, and 100 generations. The value of the maximum projection area for each iteration is shown in Fig. 4. Because GA converges to an optimum after about 90 iterations, the optimization history of 100 iterations is illustrated. The reference projection area value is calculated manually, and the pitch and yaw angle are determined in Geomagic Studio. The GA values were close to the reference value and improved over generations, as shown in Fig. 4. These results show that the GA solution is reliable and close to the maximum projection area. This method is calculated by computer and is more reliable and accurate than manual operation.

### Reference methods

To verify the performance of our system, we used two projection area measurement methods for reference as mentioned above (Fig. S3). Method 1 is photogrammetric planimetry and method 2 is the transparent grid paper method. Photogrammetric planimetry is where the auricle plane is fixed with a 1 cm2 grid paper for scale, then a photo is taken with the camera directly facing the auricle. Finally, based on the ratio of grid paper and auricle pixels shown in image-editing software (Adobe Photoshop CS5; Adobe Systems Inc., San Jose, CA, USA) we calculate the auricle projection area. The transparent grid paper method uses transparent grid paper or transparent paper (redrawn onto grid paper) to cover the auricular plane. We then outline the auricle and achieve the projection area.

### Statistical analysis

The 3D optical method was compared with the two reference methods using the paired Student’s t test, after a Kolmogorov–Smirnov test determined that the data was normally distributed. Measurement error was calculated and presented using Bland and Altman plots^24^. To assess the inter-rater reliability, the interclass correlation coefficient (ICC) with a 95% confidence interval was calculated using R (version 2.12.2). For the ICC calculations, a two-way random effect model was selected and calculated for absolute agreement of the scores, where a value of ‘1’ represented perfect agreement, and ‘0’ was interpreted as a lack of any agreement.

## Results

### Validity and reliability of measurement

**Figure 5:**
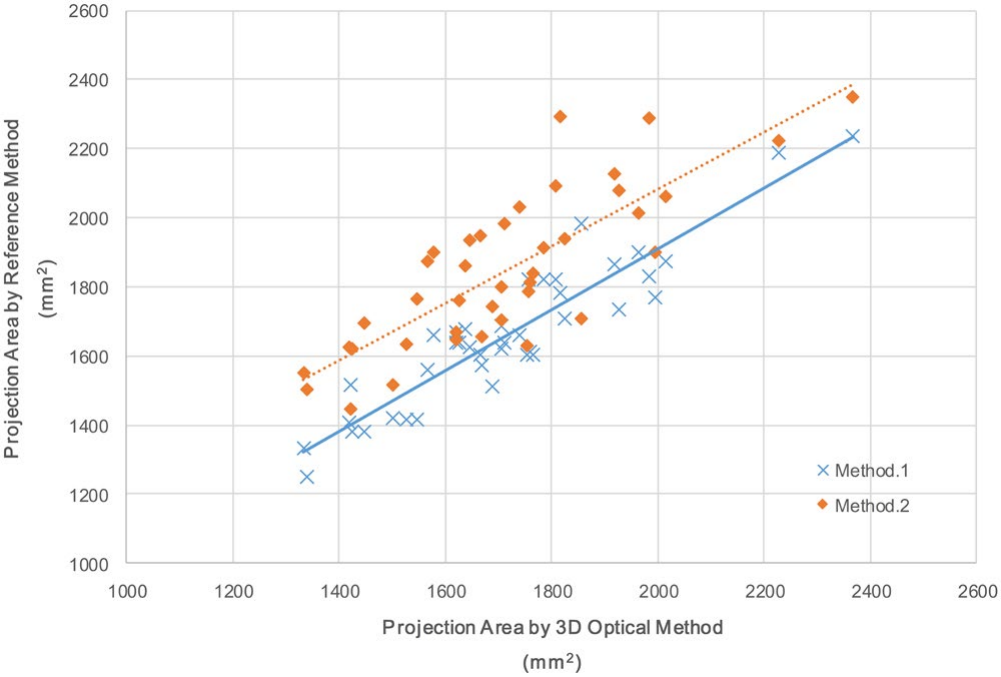
Projection area results of the 3D optical method versus methods 1 and 2 (sample size: 40).

**Figure 6:**
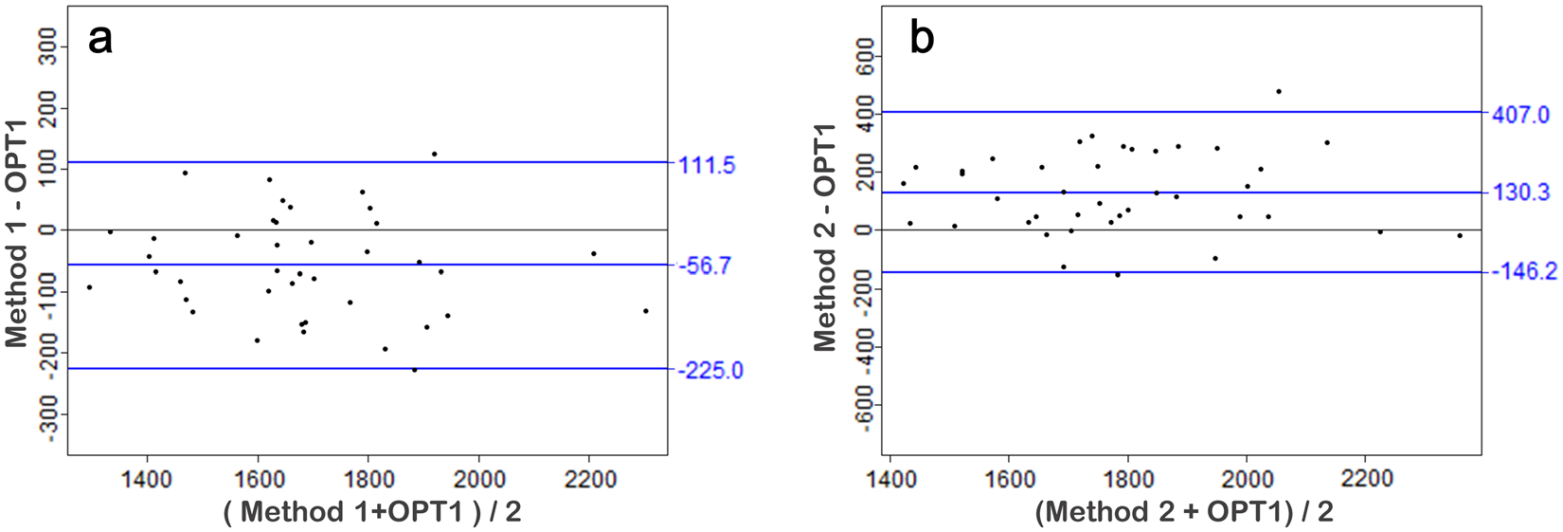
Bland and Altman comparing the 3D optical method with each reference method (sample size: 40). (**a**) 3D optical method versus method 1. (**b**) 3D optical method versus method 2.

A comparison of the auricle projection area data derived by the three methods is shown in Fig. 5 (Table S2). Using the paired Student’s t test method, the P values between the 3D optical method and methods 1 and 2 are 0.39 and 0.09, respectively. A Bland and Altman plot comparing the 3D optical method with each reference method is shown in Fig. 6. No significant difference was detected between the 3D optical method and method 1. However, the P value between the 3D optical method and method 2 is lower than it is for method 1, mainly because of the lower accuracy and reliability of method 2^17^.

All data on inter-rater reliability together with confidence intervals and P values are presented in Table 1 (further details are given in Table S3). The 3D optical method showed a good reliability ($r=0.97,\,P < 0.001$), and is clearly better than the two reference methods. The reliability of the two reference methods also match the conclusions of^17^. The 95% confidence intervals are presented in Table 1.

**Table 1:** Inter-rater reliability of three raters by ICC for auricle projection area measurement by 3D optical method and two reference methods (sample size: 40).

	Our method	Method 1	Method 2
ICC	0.97	0.85	0.70
95% CI	0.95–0.98***	0.61–0.93***	0.49–0.83***

CI = confidence interval.

***P < 0.001.

### Typical case with auricle defect

**Figure 7:**
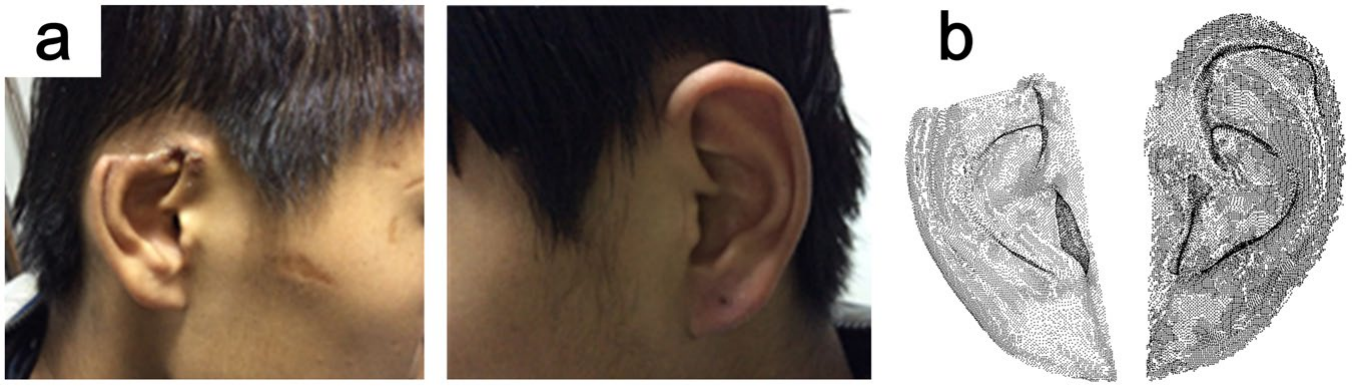
Example of an auricle defect identification and measurement case. (**a**) Photos of both ears of the participant from the side; (**b**) Point cloud result of the maximum projection area solved using the GA method

We present a typical example case identified by our 3D method. The subject is Chinese, male, 17 years old, with right auricle defects caused by trauma. Figure 7 shows the point cloud result of the maximum projection area solved using the GA method, more details were shown in Fig. S3. The measurement result is listed in Table 2. The data were validated using methods 1 and 2. Based on the result, a forensic analysis concludes that the auricle defect of the participant is 13.5%, and identifies it as a slight wound.

**Table 2:** The measurement results of Auricle defect case.

Measure Position	Maximum projection area & Defected percentage (mm^2^)			Surface area(mm^2^)
	3D method		Method 2	
Defected auricle	1588.944 (86.5%)	1545 (87.1%)	1839 (86.7%)	3656.384
Healthy auricle	1836.157 (100%)	1774 (100%)	2120 (100%)	2915.675

## Discussion

The above experiment results provide evidence that the 3D optical measurement method is a viable technique for the reliable identification of auricle defects and more repeatable than common used methods, and the automatically data process improve the accuracy of projection area. So the proposed method makes a better inter-rater reliability, and the above example case shows this system is suitable for daily medicolegal investigations.

The results of this study shows that 3D optical surface scanning is a suitable method for the documentation of auricle defects. The 3D models of the scanned auricle display high resolution and accuracy, including all fine detail. The acquisition and the electronic storage of the full-scale surface of the auricle allows for examination at any time. Data exchange between investigating forensics at national and international levels can be effected easily with electronic data carriers. The 3D models would provide great help during auricle reconstruction treatment. The 3D model data can be used to generate ear prostheses using a rapid prototyping machine and CAD/CAM technology^25,26^. Auricle surface areas can also determine the amount of grafted skin or expansion skin required for reconstruction^27^. Furthermore, this study would be also useful for other areas of forensic medicine^16,28,29^, such as the surface area determination of wounds and scars^17^.

There are several aspects that need to be further improved in the proposed measurement system. First, when we measured the normal auricles of 20 people, the results showed that the auricles on both sides of the head are not exactly the same in size (Tables S2 and S3). The mean deviation value of these samples is 4.9% (SD = 2.7%). A problem could be found in some cases, where the two ears of a person have obviously different projection areas. As a result, using the defective auricle and heathy auricle maximum projection area ratio to estimate the percentage of the defect might not be accurate enough. It would be better if the healthy auricle could be flipped and scaled to the same size as the defective auricle based on the feature points of both auricles, so that more accurate auricle defects could be identified in all situations. Second, as mentioned in Section 2 the whole processes cannot be completed automatically. Testers still need to segment the auricular edge manually, and this could introduce errors. The point cloud segmentation method for automatic auricular data extraction would be adopt to this system in the future.

This study showed that our 3D optical measurement solution is suitable for the identification of auricle defects. Most processes are completed by computer, and hence it is efficient and convenient for the documentation of identification results. Furthermore, our method is more efficient than existing methods because most of the data processing is completed automatically. We applied this system to a medicolegal investigation and obtained a favorable result for auricle defect identification.

## Electronic supplementary material

Supporting Information
