# Supplementary material for: Identification and Documentation of Auricle Defects using Three-dimensional Optical Measurements
Source: Sci Rep. 2018 Feb 12;8:2869. doi: 10.1038/s41598-018-21289-x (PMC5809514; doi:10.1038/s41598-018-21289-x)
Supplement: Supplementary file 1 — Supporting Information [file 41598_2018_21289_MOESM1_ESM.doc]

Supporting Information for the publication:

**Identification and documentation of auricle defects using three-dimensional optical measurements**

Guomin Zhan^1^; Liya Han^1^, Zhongwei Li^1^*; Zilong Liu^2^*; Jiaqi Fu^2^ and Kai Zhong^1^

^1^ State Key Laboratory of Material Processing and Die & Mould Technology, Huazhong University of Science and Technology

^2^ Department of Forensic Medicine, Tongji Medical College of Huazhong University of Science and Technology.

*Corresponding author:

E-mail: [zwli@hust.edu.cn](mailto:zwli@hust.edu.cn) (Zw L)

E-mail: [liuzilongfy@hust.edu.cn](mailto:liuzilongfy@hust.edu.cn) (Zl L)

S1 Fig. A detail view of the example auricle 3D model

S2 Fig. The reference measurement methods: (a) Photogrammetry planimetry (Method.1); (b) Transparent grid paper method (Method.2).

S3 Fig. The defected and healthy auricle 3D models of example case.

S1 Table. The developed 3D optical measurement device specification parameter

S2 Table. Comparison of auricle projection area using 3D Optical Method and two reference method by Rater 1.

S3 Table. Comparison of auricle projection area using 3D Optical Method and two reference method by Rater 2 and Rater 3.

S1 Table. The developed 3D optical measurement device specification parameter

| **Single Scan Range** | 200 × 160 – 400×320mm^2^ |
| --- | --- |
| **Single Maximum Points Amount** | 1.31million |
| **Point Cloud Density** | 0:156 – 313mm^2^ |
| **Measurement Error** | ±0.020mm |
| **Single Scan Time** | 0.1s |


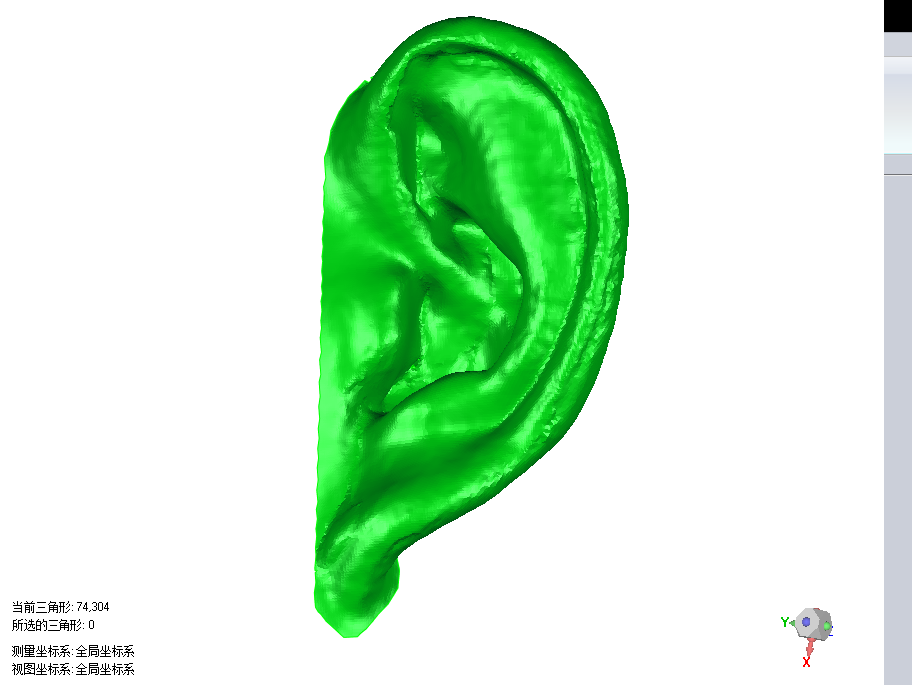


S1 Fig. A detail view of the example auricle 3D model


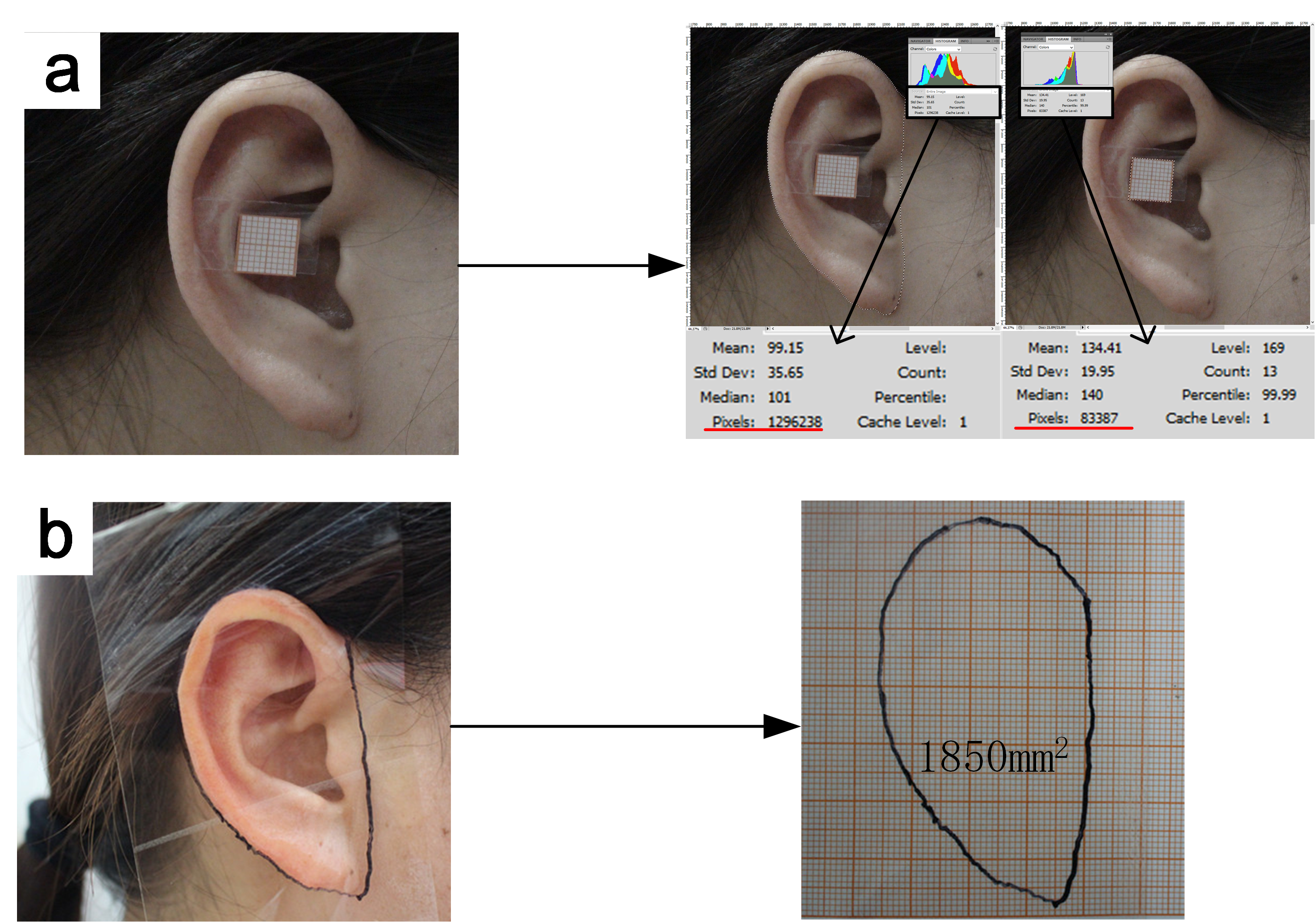


S2 Fig. The reference measurement methods:

(a)Photogrammetry planimetry (Method.1); (b)Transparent grid paper method (Method.2).


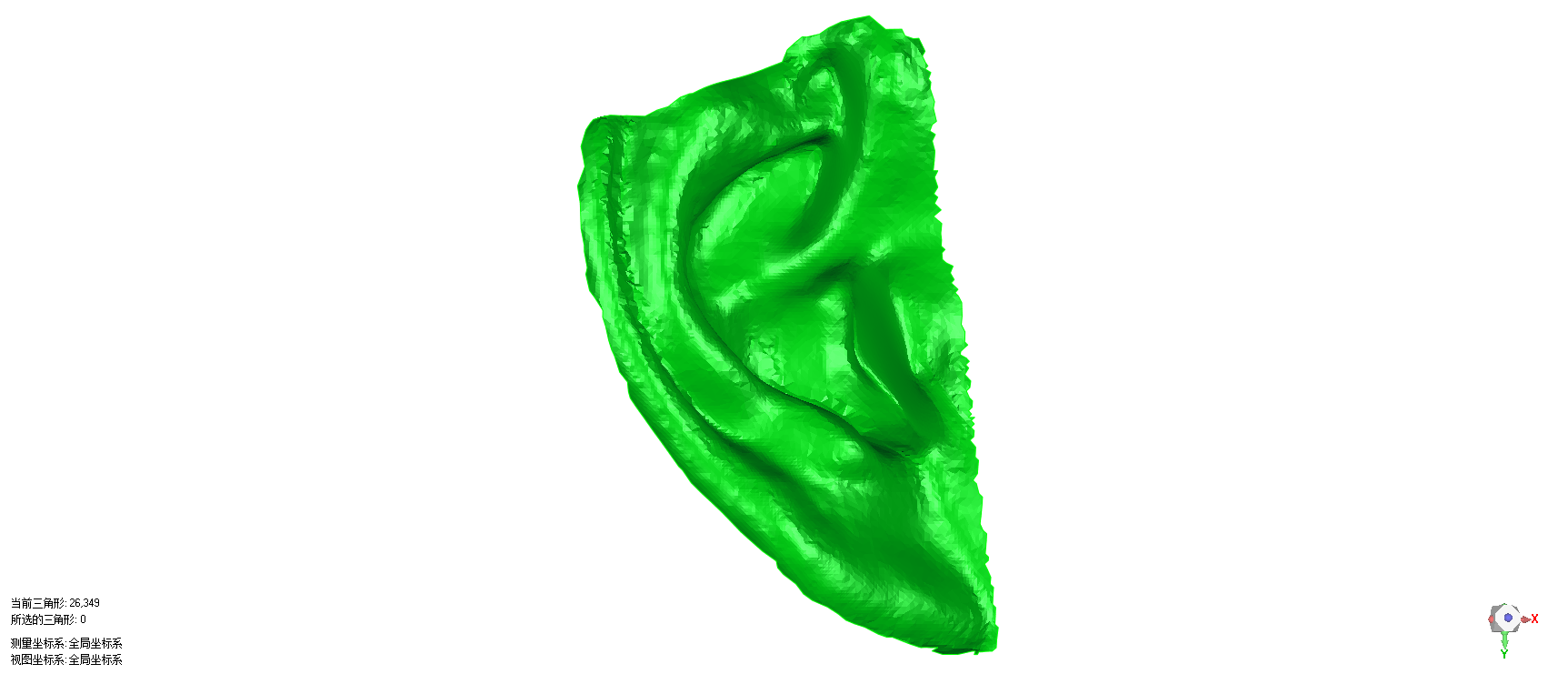

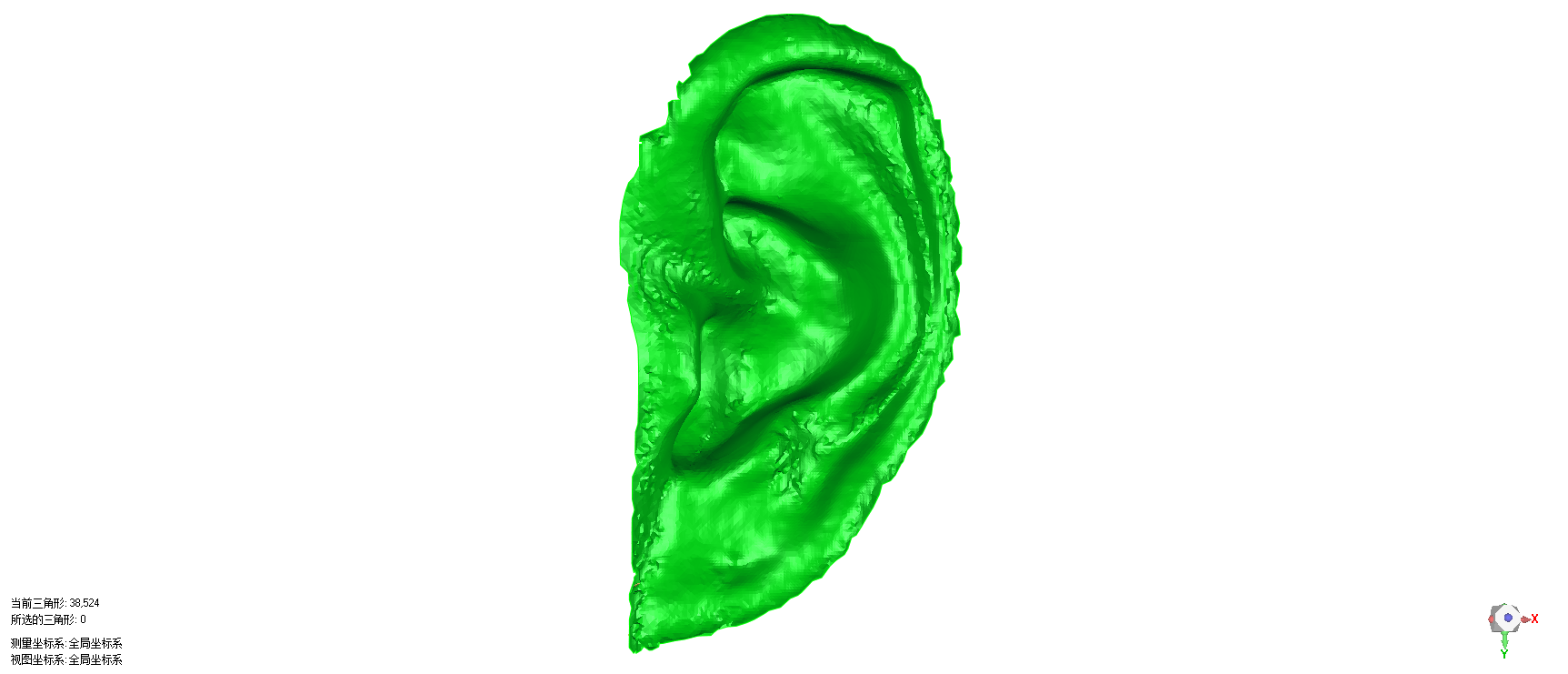


S3 Fig. The defected and healthy auricle 3D models of example case (Details of the auricle in the 3D model have a good presentation)

S2 Table. Comparison of auricle projection area using 3D Optical Method and two reference method by **Rater 1**

| **Case** | **Sex** | **Side** | **3D Optical Method** | **Reference Methods** | |
| --- | --- | --- | --- | --- | --- |
|  |  |  |  | **Method.1** | **Method.2** |
| **1** | M | L | 1647.39 | 1624 | 1937 |
|  |  | R | 1626.71 | 1640 | 1759 |
| **2** | F | L | 1639.35 | 1678 | 1860 |
|  |  | R | 1667.75 | 1602 | 1946 |
| **3** | M | L | 1566.53 | 1558 | 1873 |
|  |  | R | 1761 | 1612 | 1812 |
| **4** | F | L | 1418.77 | 1405 | 1623 |
|  |  | R | 1333.9 | 1332 | 1552 |
| **5** | M | L | 1918.83 | 1867 | 2128 |
|  |  | R | 1809.03 | 1820 | 2091 |
| **6** | F | L | 1424.11 | 1381 | 1619 |
|  |  | R | 1525.98 | 1414 | 1635 |
| **7** | M | L | 1448.99 | 1382 | 1695 |
|  |  | R | 1501.95 | 1419 | 1516 |
| **8** | F | L | 1579.1 | 1662 | 1902 |
|  |  | R | 1825.3 | 1708 | 1940 |
| **9** | M | L | 1710.68 | 1640 | 1983 |
|  |  | R | 2014.24 | 1875 | 2060 |
| **10** | M | L | 1548.12 | 1416 | 1764 |
|  |  | R | 1689.31 | 1510 | 1743 |
| **11** | F | L | 1341.3 | 1249 | 1504 |
|  |  | R | 1422.14 | 1516 | 1446 |
| **12** | F | L | 1740.24 | 1662 | 2029 |
|  |  | R | 1926.81 | 1734 | 2077 |
| **13** | F | L | 1784.96 | 1822 | 1913 |
|  |  | R | 1965.14 | 1898 | 2012 |
| **14** | F | L | 1705.93 | 1620 | 1799 |
|  |  | R | 1706.18 | 1687 | 1704 |
| **15** | M | L | 2227.28 | 2189 | 2224 |
|  |  | R | 2368.34 | 2237 | 2350 |
| **16** | M | L | 1816.33 | 1781 | 2293 |
|  |  | R | 1985.4 | 1828 | 2287 |
| **17** | M | L | 1765.97 | 1601 | 1837 |
|  |  | R | 1996.75 | 1770 | 1900 |
| **18** | F | L | 1758.5 | 1821 | 1787 |
|  |  | R | 1857.84 | 1983 | 1707 |
| **19** | F | L | 1669.46 | 1571 | 1656 |
|  |  | R | 1755.13 | 1603 | 1629 |
| **20** | F | L | 1619.74 | 1636 | 1646 |
|  |  | R | 1621.23 | 1670 | 1669 |

S3 Table. Comparison of auricle projection area using 3D Optical Method and two reference method by **Rater 2 and Rater 3**

| **Case** | | **Rater 2** | | | **Rater 3** | | |
| --- | --- | --- | --- | --- | --- | --- | --- |
|  |  | 3D Optical Method | Method.1 | Method.2 | 3D Optical Method | Method.1 | Method.2 |
| **1** | L | 1599.78 | 1573 | 1528 | 1494.36 | 1611 | 2050 |
|  | R | 1609.95 | 1544 | 1895 | 1558.91 | 1634 | 1426 |
| **2** | L | 1671.65 | 1648 | 2024 | 1720.46 | 1632 | 1815 |
|  | R | 1713.44 | 1675 | 1748 | 1665.98 | 1589 | 1606 |
| **3** | L | 1504.97 | 1541 | 2388 | 1506.94 | 1527 | 2479 |
|  | R | 1787.07 | 1655 | 2256 | 1785.53 | 1531 | 1597 |
| **4** | L | 1432.81 | 1523 | 2043 | 1406.59 | 1663 | 1558 |
|  | R | 1344.79 | 1329 | 1205 | 1304.31 | 1324 | 1559 |
| **5** | L | 1890.43 | 1847 | 2402 | 1916.33 | 1640 | 2163 |
|  | R | 1830.19 | 1741 | 1529 | 1841.57 | 1748 | 1601 |
| **6** | L | 1547.15 | 1219 | 1179 | 1570.36 | 1156 | 1268 |
|  | R | 1543.53 | 1383 | 1739 | 1460.34 | 1243 | 1839 |
| **7** | L | 1454.89 | 1272 | 1568 | 1419.39 | 1256 | 1790 |
|  | R | 1480.47 | 1334 | 1314 | 1525.47 | 1246 | 1834 |
| **8** | L | 1551.78 | 1719 | 2043 | 1558.71 | 1798 | 2351 |
|  | R | 1836.77 | 1461 | 1572 | 1912.26 | 1548 | 1791 |
| **9** | L | 1662.95 | 1575 | 1902 | 1766.72 | 1486 | 2282 |
|  | R | 2067.42 | 1607 | 1520 | 2091.19 | 1506 | 1609 |
| **10** | L | 1517.78 | 1450 | 2027 | 1428.38 | 1381 | 1656 |
|  | R | 1700.64 | 1549 | 1206 | 1669.86 | 1499 | 1486 |
| **11** | L | 1316.89 | 1146 | 1500 | 1321.88 | 1122 | 1085 |
|  | R | 1420.92 | 1241 | 1725 | 1374.18 | 1362 | 1939 |
| **12** | L | 1723.1 | 1436 | 1428 | 1695.87 | 1387 | 1668 |
|  | R | 2027.39 | 1591 | 2391 | 2159.98 | 1503 | 2275 |
| **13** | L | 1743.91 | 1467 | 1866 | 1815.58 | 1401 | 1649 |
|  | R | 1742.09 | 1515 | 1425 | 1820.14 | 1580 | 1666 |
| **14** | L | 1698.64 | 1530 | 1365 | 1662.46 | 1584 | 1213 |
|  | R | 2199.66 | 1563 | 2540 | 2266.97 | 1557 | 2352 |
| **15** | L | 2252.01 | 2083 | 2766 | 2195.26 | 2064 | 2149 |
|  | R | 2364.86 | 1941 | 2209 | 2396.31 | 1952 | 1849 |
| **16** | L | 1839.76 | 1788 | 1991 | 1882.26 | 1871 | 1774 |
|  | R | 2002.05 | 1691 | 2391 | 2112.17 | 1720 | 1894 |
| **17** | L | 1828.31 | 1459 | 1308 | 1888.1 | 1445 | 1845 |
|  | R | 2030.89 | 1425 | 2059 | 2048.36 | 1496 | 2235 |
| **18** | L | 1729.84 | 1421 | 1677 | 1790.04 | 1458 | 1720 |
|  | R | 1855.85 | 1307 | 1647 | 1709.42 | 1332 | 1509 |
| **19** | L | 1672.14 | 1199 | 2665 | 1650.41 | 1182 | 2329 |
|  | R | 1745.21 | 1256 | 1954 | 1770.34 | 1228 | 1036 |
| **20** | L | 1625.38 | 1495 | 1441 | 1689.26 | 1519 | 1429 |
|  | R | 1643.77 | 1609 | 1697 | 1673.69 | 1675 | 1372 |
